# A qualitative description of telemedicine for acute stroke care in Norway: technology is not the issue

**DOI:** 10.1186/s12913-014-0643-9

**Published:** 2014-12-19

**Authors:** Tove Sørensen, Kari Dyb, Ellen Rygh, Rolf Salvesen, Lars Thomassen

**Affiliations:** Norwegian Centre for Integrated Care and Telemedicine, PO Box 35, NO-9038 Tromsø, Norway; Norwegian Centre for Integrated Care and Telemedicine, Kirkeveien 9, NO-4816 Kolbjørnsvik, Norway; Nordland Hospital, NO-8092 Bodø, Norway; University of Tromsø, 9038 Tromsø, Norway; Haukeland University Hospital, NO-5021 Bergen, Norway

**Keywords:** Telestroke, Technology, Qualitative, Overview, Norway, Acute stroke, Collaboration

## Abstract

**Background:**

To assist small hospitals in providing advanced stroke treatment, the Norwegian Directorate of Health has recommended telemedicine services. Telestroke enables specialists to examine patients via videoconferencing supplemented by teleradiology and to provide decision support to local health care personnel. There is evidence that telestroke increases thrombolysis rates.

In Norway, telemedicine has mainly been used in non-critical situations. The first telestroke trials took place in 2008. The aim of this paper is to present an overview of telestroke trials and today’s status with telestroke in Norway. Based on the divergent experience from two health regions in Norway, the paper discusses crucial factors for the integration of telestroke in clinical practice.

**Methods:**

This is a descriptive study based on multiple methods to obtain an overview of the practice and experience with telestroke in Norway. A Web and literature search for ‘telestroke in Norway’ was performed and compared with a survey of telemedicine services at the country's largest hospitals. These findings were supplemented by interviews with key personnel involved in telestroke in two of four health regions, as well as hospital field observations and log data of telestroke transmissions from five of the hospitals involved.

**Results:**

In Norway, experience in telemedicine for acute stroke care is limited. At the beginning of 2014, three of four regional health authorities were working with telestroke projects and services. Integration of the service in practice is challenging, with varying experience.

The problems are not attributed to the technology in itself, but to organization (availability of staff on duty 24/7 and surveillance of the systems), motivation of staff, logistics (patient delay), and characteristics of the buildings (lack of space).

**Conclusions:**

Prerequisites for successful integration of telestroke in clinical practice include realization of the *collaboration potential* in the technology with consistent procedures for training and triage, availability of the equipment, and providing advice beyond questions concerning thrombolysis.

## Background

Norway has been one of the pioneers in telemedicine and e-health (information and communication technologies in health, ICT) along with Northern America and Australia [1-3]. In 1997 the first in a series of national action plans for ICT in the health and social sector, ‘More health for each bIT’, was launched [4]. Reimbursement for telemedicine was introduced back in 1996 [4]. Thus, the foundation for telemedicine and e-health has been in place for several years: Secure infrastructure (Norwegian Health Net), governance, some financial mechanisms and a sound knowledge base from the many pilot projects and the research in the field. With a few exceptions, telemedicine in Norway has mainly been used in non-critical situations.

Stroke is the third most frequent cause of death in Norway, and the most common cause of severe disability. ‘Time is brain’ [5]: In stroke treatment, thrombolysis must be started within 4.5 hours of symptom onset, the ‘thrombolysis window’. Besides rapid transport to hospital, it is vital to make a reliable diagnosis as quickly as possible when patients with suspected stroke are admitted to hospital. To supplement local hospitals’ expertise and experience, organization in a telestroke service has proved effective [6,7]. In this article, ‘telestroke’ implies emergencies where doctors in central stroke units examine patients via videoconferencing (VC), have online access to imaging diagnostics (most often CT), and discuss diagnosis and treatment with local health staff. Meta-analyses show that teleconsultations are comparable with face-to-face consultations and contribute to increase thrombolysis rates [8,9]. During the past 10–15 years, telestroke use has increased in North America and Europe [10-13]. A telestroke service between Helsinki University Hospital and five local hospitals led to increased thrombolysis rates [14]. Apart from this, documentation of telestroke experience in the Nordic countries is scant.

A stated objective in Norway is that all 5 million inhabitants should have equal access to health services regardless of where they live. Long distances with fjords, mountains, and a harsh climate make travel to a specialized hospital a challenge, even with a publicly funded, decentralized hospital structure. Norway should therefore be a highly suitable country for telemedicine in general and telestroke in particular. Telemedicine is recommended by the Norwegian Directorate of Health in the 2010 national guidelines for stroke treatment to enable smaller hospitals to provide advanced stroke treatment [15]. An objective specified in the description of services to be provided by the hospital trusts in 2012 is that 20% of patients with cerebral infarction under the age of 80 should be treated with thrombolysis. In 2011 the national average was 7.8% and in 2012 it was 9.4%, where the Western region stands out from the others (Table 1) [16].

**Table 1:** **Thrombolysis rate (%) in Norway age 18-80 years per health region (1 January 2011 - 31 August 2013)**

**Name**	**Freq**	**CI**
Country	9.59	9.12 - 10.1
Central Norway	7.43	6.43 - 8.57
Northern Norway	6.32	5.21 - 7.64
South-Eastern Norway	9.83	9.19 - 10.5
Western Norway	14.9	13.6 - 16.4
Private	2.65	1.63 - 4.24

In Norway, the first telestroke trials took place in 2008. As there is no systematic report on the experience in the field, the aim of this paper is to present an overview of telestroke trials and today’s status with telestroke in Norway. Based on the divergent experience from two health regions in Norway, one succeeding and one failing to integrate a telestroke service, we will discuss crucial factors for the integration of telestroke in clinical practice.

## Methods

This is a descriptive study of telestroke in Norway, presenting (i) an overview of the hospitals where telestroke has been (tried) implemented; and (ii) some hospitals’ specific experience with telestroke. For the overview and status (i), a Web and literature search for ‘telestroke in Norway’ was performed and compared with a survey of telemedicine services at the country's largest hospitals from 2012 [17]. Based on this knowledge, the ‘snowball method’ [18] was used to contact and interview people who had experience with telestroke in acute situations. In the snowball method, information from interviews is used to get in touch with new potential respondents. These interviews were conducted by telephone and e-mail by three of the five authors. Since Norway is a small country with a transparent telemedicine community, we found the method sufficient to ensure an overview of the field.

To obtain in-depth understanding of health personnel’s experience, including non-technological challenges with the use of telestroke in Norway, part (ii) includes log data of telestroke transmissions, hospital observations and interviews with key personnel from two of the four health regions. These regions were selected based on their experience with telestroke services, successes and failures respectively. The data comprise of one week of field observations from three hospitals, log data analysis, and interviews lasting 30–90 minutes with 23 hospital doctors and stroke nurses in the five hospitals involved. In addition, two of the authors have first-hand experience with telestroke in their regions. The study has been approved by the Regional Committee for Medical and Health Research Ethics (REK Nord, 2010/2277).

## Results

**Figure 1:**
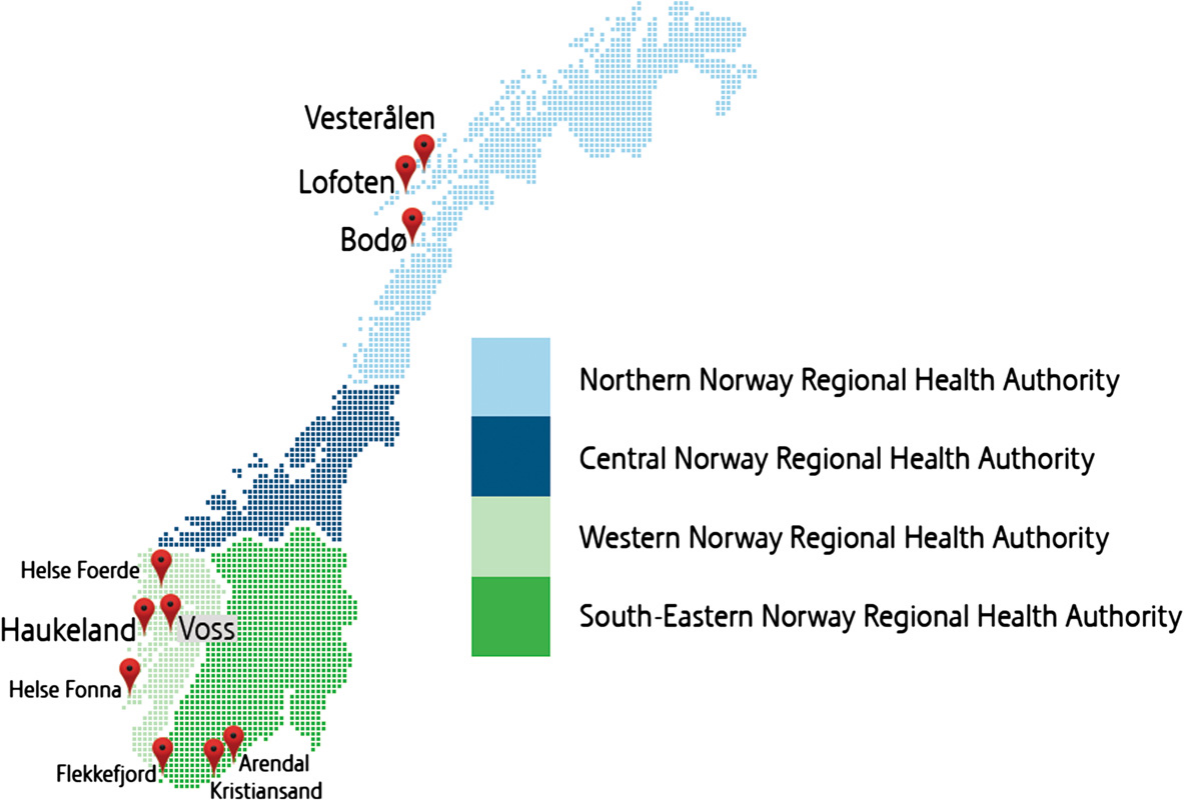
**Hospitals with telestroke in Norway by Jarl-Stian Olsen, NST.**

There is no national systematic recording of telestroke consultations in Norway. At the end of January 2013, three of four regional health authorities had, or had tried telestroke (Table 2 and Figure 1). Telestroke was used in acute stroke management for assessment of differential diagnostics, thrombolytic therapy, triage and transfer of expertise.

**Table 2:** **Overview of telestroke in three Regional Health Authorities (RHA) in Norway (1 January 2014)**

**Region**	**Collaborating hospitals**	**Technology**	**Status**	**Telestroke operation**
Western Norway RHA	Health Bergen	Two-way video-conferencing and online X-ray transmission	Pilot 2008	24/7
			Routine service from 2009	
	Haukeland University Hospital			
	● Voss Hospital			
	Health Fonna	Two-way video-conferencing and online X-ray transmission	2012	Mo-Fr -> 20:00
				Sa-Su -> 16:00 If local staff on call is at home, they may connect to Haukeland Univ Hospital
	Haugesund Hospital			
	● Stord Hospital			
	● Odda Hospital	ᅟ		
	Health Førde		2009	Not in use due to technical problems
	Førde Central Hospital			
	● Nordfjord Hospital			
	● Lærdal Hospital			
South-Eastern Norway RHA	Sørlandet Hospital	Web/PC- based solution in Kristiansand. VC on patients’ side. Online X-ray transmission	2012 in Flekkefjord January 2013 in Arendal	24/7
	Kristiansand Central Hospital			
	● Flekkefjord Hospital			
	● Arendal Hospital			
Northern Norway RHA	Nordland Central Hospital	Two-way video-conferencing and online X-ray transmission	Pilot-project 2010-2012	Mo-Fr -> 19:30
	Nordland Central Hospital, Bodø		Lofoten Hospital used service untill the end of the project. Not used in Vesterålen.	
	● Lofoten Hospital			
	● Vesterålen Hospital			

All sites used technologies (systems) according to Norwegian regulations.

In the Western region, three health trusts have experience with telestroke. Haukeland University Hospital (HUS) established telestroke with the local hospital Voss as a pilot project in 2008. The service has been in routine operation since 2009. The teleconsultations were recorded consecutively. During the period 2008–2012, 53 telestroke consultations were performed, of which 33 resulted in thrombolysis. One teleconsultation was unsuccessful for technical reasons, and one concerned the acute patient pathway after the thrombolysis window. Voss hospital has about 70 stroke cases per year, and reported that the number of thrombolysis rose from zero to about eight cases per year after the hospital was connected with telestroke. In 2011–2012, the thrombolysis rate was 13% at Voss and 15% at HUS [16].

When a patient arrived at the Voss local hospital with suspected stroke, the department of neurology at HUS was contacted while the patient was sent for a computed tomography (CT) scan. A radiologist assessed the images, and the neurologist went to a dedicated telestroke studio with VC equipment and online access to the CT images to assist in the assessment of the patient. Telestroke was used for all patients with symptoms of acute stroke, including those outside the thrombolysis window. It was estimated that telestroke took 3–4 minutes longer than a telephone consultation. If technical problems occurred, the equipment was restarted. Telephone was used if the restart did not work. The specific stroke treatment was sometimes changed during the telestroke conference. Although the doctors at the local hospital were mostly confident about the treatment, they reported that telestroke helped them to keep up to date with current stroke treatment. All new doctors received telestroke training. Senior doctors at both central and local site described the service as good, and emphasized the following success criteria for telestroke: Motivated staff, radiologists and neurologists on call 24/7, teleradiology by a common radiology network, and a low threshold for contacting the department of neurology at the central hospital. They reported that the challenge was unclear responsibilities for technical and financial maintenance of the communication lines.

In the Western region, two other telestroke services were tried established: one (Health Fonna) was recently established and therefore had limited experience with the service; the other (Health Førde) introduced telestroke between the central hospital and two local hospitals in 2009. The second service was never put into operation due to unresolved allocation of responsibility related to ICT operations and networks.

In the Northern region, a telestroke project was launched after the volcanic eruption in Iceland in April 2010, which paralysed air ambulance services. Telestroke was established between the department of neurology at the central hospital in Bodø and two local hospitals on the archipelago, Lofoten and Vesterålen (Figure 1). The equipment was of the same type as used in emergency departments in Northern Norway, the ‘VAKe’ model [19]. In 2010, health staff at all hospitals was trained in the use of the telestroke equipment and procedures for patients presenting with acute stroke were revised to achieve consistency. All three hospitals reported that the technology (VC and teleradiology) functioned satisfactorily. However, from September 2010 to February 2013, only four telestroke consultations took place from Lofoten to Bodø, none from Vesterålen. Vesterålen Hospital had a neurologist available during office hours in addition to local doctors. The hospital reported that local qualifications were sufficient, and that there was consequently no need for the telestroke service.

Telestroke consultations were offered only during the periods when the second-call neurologist was on duty in-house at the central hospital in Bodø (8:00–19:00). When a patient with suspected stroke arrived at the local hospital, the local doctor decided whether to contact the neurology department, telephoned the neurologist on-call, and connected the VC equipment. Telestroke was the preferred choice at Lofoten hospital when thrombolysis was relevant. Health staff at the local hospital reported that they found telestroke a better service than telephone and requested the service extended to 24/7 availability. However, the central hospital found that the patient volume was too low for the service to be feasible when decisions needed to be made instantly.

At Lofoten hospital, the telestroke equipment was initially placed in the emergency department, then moved because of lack of space, so that it had to be taken out from storage and connected when needed. This was described as a disadvantage in a situation under time pressure. At Vesterålen hospital, the telestroke unit was placed in the intensive care unit due to limited space, and not in the emergency department where the patient first arrived. Here too, the availability of the equipment was described as cumbersome and time-consuming. In Bodø, the neurologist had to move to another wing of the hospital for teleconsultations.

In the Southern region, Kristiansand Central Hospital established telestroke with a local hospital (Flekkefjord) in the second half of 2012. Since 2007, this local hospital had carried out 40–50 thrombolysis treatments with telephone-based consultation and teleradiology. The central hospital chose a PC-based VC-system allowing the neurologist to examine the patient from any office, which saved time and streamlined the work procedures. The image quality was described as satisfactory. The disadvantage was that the local hospital could not see the stroke specialist. At the local hospital, the telestroke conference took place in the emergency department before the patient was sent for CT. The procedure was that all stroke patients should have telestroke, including those who were not candidates for thrombolysis. The same PC-based solution was introduced in another local hospital (Arendal) in early 2013, when their after-hours neurologist service was discontinued. Here the telestroke equipment was installed in the radiology department, where the patient was examined on the CT table via a fixed wall-mounted camera. It is too early to comment on the experience from the Southern region.

## Discussion

Although Norway has extensive experience with telemedicine in general, there is limited experience with telestroke and the volume is low compared to international experience. Few services have been operative over time and are integrated in clinical practice. Haukeland University Hospital is an exception, where telestroke was started in 2008 and has been in routine use since 2009.

International telestroke networks have established requirements for participating centres to consult the stroke centre in connection with specific indications, including arrival at the hospital within the thrombolysis window, reduced level of consciousness, progressive strokes, brainstem symptoms, or cerebral haemorrhage [20,21]. The Norwegian telestroke networks had no criteria for mandatory telestroke consultation. It is possible that atypical cases have not been discussed via telestroke, which may explain the low frequency of telestroke consultations. This may mean that patients with ‘minor’ or ‘atypical’ strokes did not receive an optimal treatment. The fear of treating ‘non-stroke’ with thrombolysis may also have resulted in inadequate treatment of true cerebral infarction [21].

International literature reports that telestroke results in an increased thrombolysis rate [10]. In Finland, the thrombolysis rate was 57.2% for patients who received teleconsultations [14]. Such parameters were not recorded systematically in our study, except for Voss Hospital, which reported that the thrombolysis rate rose quickly after the hospital started using telestroke. The Western region has a significantly higher thrombolysis frequency than the rest of the country (Table 1). As the region geographically is as challenging as most parts of the country, we can assume that a long-term focus on both thrombolysis and telestroke may have been contributing factors. The cumulated thrombolysis frequency in the region was 15% in the period 2011–2013. The region has now established telestroke in all local hospitals. The regional health authorities have stated that departments with good stroke collaboration should be able to provide thrombolysis therapy for about 20% of all patients with ischemic stroke and 40-50% of those who arrive within 4.5 hours [22].

A major challenge is that the patients arrive too late at the hospital, or the time of symptom onset is unknown. In Bodø Hospital this applied to more than 80% of the patients with cerebral infarction [23]. It is not clear to what degree the variation in thrombolysis rates in Norway is due to differences in patient delay or to the doctors’ attitude to thrombolytic therapy in patients with mild strokes [23].

In this study few technical problems were reported related to the equipment or transmissions, but there were unresolved allocation of responsibility related to ICT operations and networks. Other logistics were more challenging, especially the location of the VC-equipment in the hospital. If telestroke involved an extra ‘stop’ for the patient, the service was perceived as impractical. At the hospitals in the Northern region, the lack of space in the emergency department reduced the availability and accessibility of the VC-equipment. Haukeland hospital had a dedicated, spacious telestudio within the neurological department, while the Southern region chose PC-based systems to reduce the space requirements.

Motivation, or lack of it, has been a key issue in adapting telemedicine [24,25]. This might have been the case at Vesterålen Hospital, which reported having adequate local expertise and that telestroke did not represent a benefit for the hospital. Use of telestroke must be perceived as improving the *quality of the treatment:* Voss Hospital did not use telestroke when locum staff was on duty during holiday periods at the central hospital. Correspondingly, in the northern region, Vesterålen Hospital expected an experienced neurologist for telestroke consultations, not a resident doctor. Telemedicine networks where specialized regional or central stroke centres collaborate with local hospitals, can meet this request for expertise 24/7 [6,14].

At Voss Hospital, the doctors reported that it was important ‘to keep the technology warm’ and to use telestroke even when thrombolysis was not considered relevant. Regular testing and use of the equipment may be crucial for efficient use in an emergency setting. Using the VC-system for medical emergencies besides stroke, might also lower the threshold for use and thus increase the volume of telestroke.

Telemedicine in connection with acute conditions has received increased attention in recent years [11,19]. In the Northern region, it was assumed that telestroke could help small local hospitals to maintain their emergency preparedness. However, telestroke can also be used as a substitute for neurologist coverage by local hospital staff outside office hours, as in the on-call telestroke service recently introduced in Arendal Hospital in the southern region. In this way, telestroke may imply a decentralizing and a centralizing function in different contexts which may explain conflicting perceptions of the value of such services in Norway.

Today, the boundaries between telephony and video are shifting and overlapping [26]. Good video on PCs and smartphones may mean more accessible and time-saving solutions for telestroke. We will still argue that the critical factors are the non-technical issues. If telestroke shall succeed as a collaborative tool linking disciplines and departments together, it is crucial to focus on the implementation processes in the organization. Both local and central hospitals must feel that they are part of a professional community for the best treatment of the patient. Doctors at the local site must have a low threshold for contacting stroke expertise, and experience working as equal partners in a team. This was one of the explanations for the success of telestroke in the Western region.

### Limitations of the study

In this study, we have concentrated on telestroke as a visual service (two-way sound-image) and we have not surveyed telephone-based consultation. One of the four health regions has chosen *not* to use VC-based telestroke, but 24/7 telephone consultation and teleradiology between the central and the local hospitals in the region.

We have referred to the national quality indicator registry for thrombolysis treatment which shows regional differences in Norway. However, it is outside the scope of this paper to analyse the relationship between telestroke and thrombolysis frequency.

## Conclusions

Telestroke has been introduced in Norwegian hospitals since 2008. Few services have been operative over time and are integrated in clinical practice. Experience with telestroke is limited and divergent, and recordings are inadequate. Barriers to telestroke integration in the clinic are not attributed to the technology per se, except for the unresolved allocation of responsibility for ICT systems and networks, and the need for 24/7 technical support. Other equally important barriers are: Common and mandatory procedures for telestroke beyond questions about thrombolysis are needed. On site, the telestroke system must be placed where it is easily accessible and ready for use. Last, but not least, the motivation of staff is crucial to ensure a joint understanding of the quality assurance component of consulting colleagues by telestroke.

## References

[1] Elford RD (1997). Telemedicine in northern Norway. J Telemed Telecare.

[2] Gammon D, Bergvik S, Bergmo T, Pedersen S (1996). Videoconferencing in psychiatry: A survey of use in northern Norway. J Telemed Telecare.

[3] Johnsen E, Breivik E, Myrvang R, Olsen F (2006). Gevinster av norsk telemedisin.

[4] *More health for each bIT*. Oslo: Ministry of Health; 1996, https://www.regjeringen.no/nb/dokumenter/mer-helse-for-hver-bit/id87401/?regj_oss=10.

[5] Saver J (2006). Time is brain - quantified. Stroke.

[6] Audebert H (2006). Telestroke: effective networking. Lancet Neurol.

[7] LaMonte M, Bahouth M, Hu P (2003). Pathan Mea. Telemedicine for acute Stroke. Stroke.

[8] Deshpande A, Khoja S, McKibbon A, Rizo C, Jadad AR (2008). Telehealth for acute stroke management (Telestroke): Systematic Review and Environmental Scan [Technology overview 37].

[9] Schwamm LH, Holloway RG, Amarenco P, Audebert HJ, Bakas T, Chumbler NR, Handschu R, Jauch EC, Knight WA, Levine SR, Mayberg M, Meyer BC, Meyers PM, Skalabrin E, Wechsler LR (2009). A Review of the Evidence for the Use of Telemedicine Within Stroke Systems of Care: A Scientific Statement From the American Heart Association/American Stroke Association. Stroke.

[10] Müller-Barna P, Schwamm LH, Haberl RL (2012). Review. Telestroke increases use of acute stroke therapy. Curr Opin Neurol.

[11] Silva GS, Farrell S, Shandra E, Viswanathan A, Schwamm LH (2012). The status of telestroke in the United States: a survey of currently active stroke telemedicine programs. Stroke.

[12] Khan K, Shuaib A, Whittaker T, Saqqur M, Jeerakathil T, Butcher K, Crumley P (2010). Telestroke in Northern Alberta: a two year experience with remote hospitals. Can J Neurol Sci.

[13] French B, Day E, Watkins C, McLoughlin A, Fitzgerald J, Leathley M, Davies P, Emsley H, Ford G, Jenkinson D, May C, O’Donnell M, Price C, Sutton C, Lightbody C (2013). The challenges of implementing a telestroke network: a systematic review and case study. BMC Med Inform Decis Mak.

[14] Sairanen T, Soinila S, Nikkanen M, Rantanen K, Mustanoja S, Färkkilä M, Pieninkeroinen I, Numminen H, Baumann P, Valpas J, Kuha T, Kaste M, Tatlisumak T, Finnish Telestroke Task Force (2011). Two years of Finnish Telestroke: thrombolysis at spokes equal to that of the hub. Neurology..

[15] *National guidelines for treatment and rehabilitation for ischemic stroke Norwegian Directorate of Health 2010*, IS-1688, http://www.helsedirektoratet.no/publikasjoner/nasjonal-retningslinje-for-behandling-og-rehabilitering-ved-hjerneslag-fullversjon/Sider/default.aspx.

[16] Quality indicators for the health service. Oslo: Norwegian Directorate of Health; 2013 [cited 2014 20 January]; Available from: http://helsenorge.no/Helsetjenester/Sider/Kvalitetsindikatorer-rapporter.aspx?kiid=Trombolysebehandling_ved_hjerneinfarkt.

[17] Zanaboni P, Knarvik U, Wootton R (2014). Adoption of routrine telemedicine in Norway: the current picture. Global Health Action.

[18] Mosby. Mosby's Medical Dictionary. Mosby's Medical Dictionary. 8 ed2009. Elsevier. ISBN 978-0-323-05290-0.

[19] Bolle S, Larsen F, Hagen O, Gilbert M (2009). Video conferencing versus telephone calls for team work across hospitals: a qualitative study on simulated emergencies. BMC Emerg Med.

[20] Audebert HJ, Schenkel J, Heuschmann P, Bogdahn U, Haberl RL (2006). Effects of the implementation of a telemedical stroke network: the Telematic Pilot Project for Integrative Stroke Care (TEMPiS) in Bavaria, Germany. Lancet Neurol.

[21] Guerrero W, Savitz S (2013). Tissue-type plasminogen activator for stroke mimics. Continuing to be swift rather than delaying treatment to be sure. Stroke.

[22] Authority WNRH. *Treatment and rehabilitation of ischemic stroke in Western Norway Regional Health Authority*. Bergen, Norway: 2012. http://www.helse-vest.no/aktuelt/rapporter/Documents/Regionale%20planar/Regional%20plan%20-%202012-06%20Behandling%20og%20rehabilitering%20ved%20hjerneslag.pdf.

[23] Salvesen R, Eldøen G (2013). Treatment of cerebral infarction at the stroke unit in Bodø 2011–12. Tidsskr Nor Laegeforening.

[24] Chau P, Hu P (2002). Investigating health care professionals’ decisions to accept telemedicine technology: an empirical test of competing theories. Inform Manage.

[25] Walter Z, Lopez M (2008). Physician acceptance of information technologies: Role of perceived threat to professional autonomy. Decision Support Systems.

[26] Demaerschalk BM (2011). Telemedicine or telephone consultation in patients with acute stroke. Curr Neurol Neurosci Rep.

